# Pancreatic tumor in type 1 autoimmune pancreatitis: a diagnostic challenge

**DOI:** 10.1186/s12885-019-6027-0

**Published:** 2019-08-16

**Authors:** Pei Xiang, Xiaoling Zhang, Chaoyang Wang, Yuejiao Lang, Ling Xu, Li Huang, Jingxian Shen, Shi-Ting Feng

**Affiliations:** 1 Department of Radiology, The First Affiliated Hospital, Sun Yat-sen University, 58 Zhongshan 2nd Road, Guangzhou, Guangdong 510080 People’s Republic of China; 2 Department of Pathology, The First Affiliated Hospital, Sun Yat-sen University, 58 Zhongshan 2nd Road, Guangzhou, Guangdong 510080 People’s Republic of China; 3 Faculty of Medicine and Dentistry, University of Western Australia, Perth, Australia; 4 Department of Pancreatobiliary Surgery, The First Affiliated Hospital, Sun Yat-sen University, 58 Zhongshan 2nd Road, Guangzhou, Guangdong 510080 People’s Republic of China; 5 Department of Radiology, State Key Laboratory of Oncology in South China, The Cancer Center, Sun Yat-sen University, 651 Dongfeng East Road, Guangzhou, Guangdong 510060 People’s Republic of China

**Keywords:** Type 1 autoimmune pancreatitis, Pancreatic tumor, CT, MRI

## Abstract

**Background:**

The co-occurrence of type 1 autoimmune pancreatitis (AIP) and pancreatic tumor (PaT) has been previously reported. Pure AIP cases have favorable prognosis and are primarily treated with steroids, while AIP cases with PaT are associated with poor prognosis where the primary management is pancreatic resection. However, it’s a challenge to timely identify the concurrent PaT in AIP because of their similar clinical and radiological manifestations.

**Methods:**

We retrospectively reviewed the data in two medical centers from January 2010 to April 2019. The inclusion criteria were as follows: 1) completion of abdominal CT imaging before invasive procedures to the pancreas, 2) a final diagnosis of type 1 AIP using the 2011 international consensus diagnostic criteria, 3) follow-up duration of at least one month unless AIP and PaT were identified simultaneously. The presence of PaT in AIP was made based on histopathological confirmation, and the absence of PaT in AIP was defined as no pathological or radiological evidence of concurrent PaT. Clinical and radiological characteristics including gender, age, surveillance period, serum IgG4 and Ca-199 levels, biopsy, extrapancreatic involvement, CT and MR (if performed) imaging characteristics were compared between AIP with and without PaT. The Fisher’s exact test was used for qualitative variables, and nonparametric Mann-Whitney test for quantitative variables. A *p* value ≤0.05 was considered statistically significant.

**Results:**

A total of 74 patients with type 1 AIP were included, of which 5 (6.7%) had the concurrent PaT. The subtypes were pancreatic ductal adenocarcinoma (3/5), solitary extramedullary plasmacytoma in the pancreas (1/5) and cholangiocarcinoma in the pancreatic segment (1/5), respectively. Gender (*p* = 0.044), the pattern of pancreatic enlargement (*p* = 0.003), heterogeneity (*p* = 0.015), low-density (*p* = 0.004) on CT and rim enhancement on MRI (*p* = 0.050) differed significantly between AIP with and without PaT. None of the low-density characteristics on CT or other assessed MRI characteristics could significantly differentiate the two groups (p>0.05).

**Conclusions:**

Female, focal pancreatic enlargement, pancreatic heterogeneity, low-density on CT and rim enhancement on MRI are suggestive of the concurrent PaT in type 1 AIP. The characteristics of low-density on CT or other MRI characteristics did not provide further diagnostic values.

## Background

Autoimmune pancreatitis (AIP) was first proposed as a clinical entity by Yoshida et al. in 1995 [1]. Current consensus suggests AIP encompasses two different types with distinct histological and clinical profiling. Type 1 AIP is recognized as a pancreatic manifestation of IgG4-related systemic disease. Histologically, it is termed lymphoplasmacytic sclerosing pancreatitis (LPSP) or AIP without granulocyte epithelial lesions (GELs). It is characterized by periductal infiltration of lymphocytes, storiform fibrosis, abundant IgG4-positive plasma cells, and obliterative phlebitis. Clinically, predominance has been observed in elderly males, with elevated serum IgG4 levels, accompanying extrapancreatic involvement (like bile ducts, salivary glands, retroperitoneum, kidneys etc.), and an exclusive response to steroids [2–5]. The prognosis is considered favorable. The long-term survival of type 1 AIP patients has been proved to be comparable to the age- and gender-matched subjects from the general population [4]. The treatment primarily involves inducing remission and reducing relapse with steroids or sometimes immunosuppressants [6, 7].

The synchronous and metachronous occurrences of type 1 AIP and pancreatic tumors (PaT) have been previously reported. In AIP patients, the occurrence rate of PaT is about 0.1–4.8% [5–12]. This co-occurrence suggests that the definitive diagnosis of AIP cannot rule out the concurrent presence of PaT. When PaT is identified in AIP patients, management changes to diagnostic and therapeutic workup for cancer, where the principal therapy is surgical resection accompanied by chemotherapy, radiation therapy or targeted medical treatment. The prognosis would be variably poorer than that of pure AIP cases, depending on the subtype, stage and therapy choice of the concurrent PaT [13, 14]. However, AIP shares similar clinical and radiological manifestations with PaT where diagnosis can be challenging. This includes: abdominal pain, obstructive jaundice, weight loss, elevation of serum IgG4 and CA-199 levels, mass formation in pancreas, pancreatic atrophy, narrowing of main pancreatic duct (MPD) and common bile duct (CBD), decreased enhancement compared to normal pancreas, and peripancreatic vessel stricture [5, 15]. These features may obscure the diagnosis of concurrent PaT in patients with AIP, which subsequently lead to the delay of appropriate therapy. As far as we know, several studies have attempted to increase the detection of concurrent tumor in AIP, but many non-PaT tumors were also studied [8, 12, 16]. Only one study has investigated the difference between AIP patients with and without PaT, where no significant difference was observed [8]. The purpose of this study was to further evaluate the clinical and radiological markers with the aim to increase the detection of concurrent PaT in type 1 AIP.

## Methods

### Patients

**Fig. 1:**
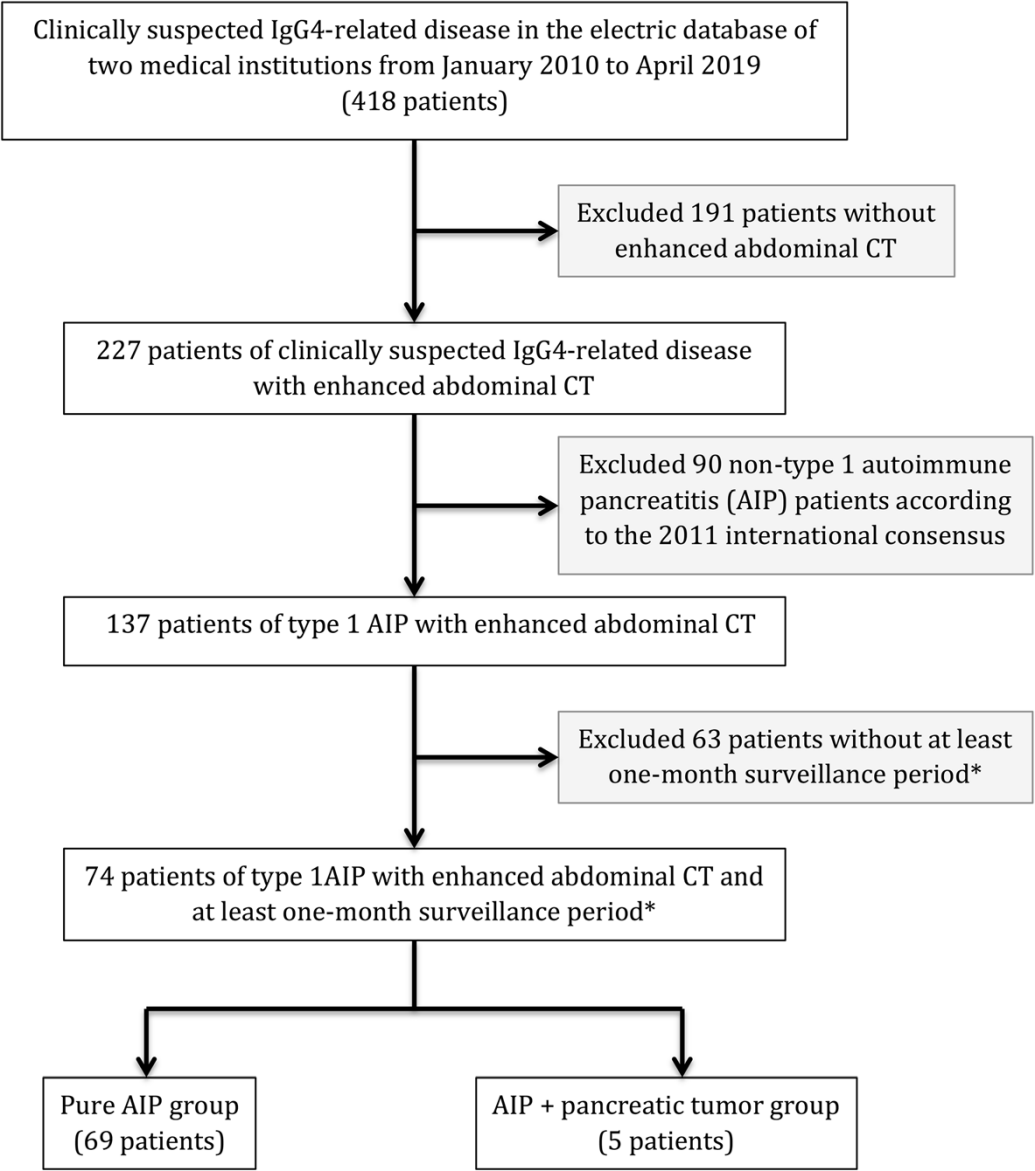
Flow diagram of study population. * If type 1 autoimmune pancreatitis and pancreatic tumor were identified at the same time, one-month surveillance period was not required for patient inclusion

The study proposal was reviewed by the ethical committee of our institution where the need for informed patient consent was waived. Patient data from two medical institutions between January 2010 and April 2019 were retrospectively reviewed. The inclusion criteria were as follows: 1) completion of dual-phase enhanced abdominal CT imaging before any invasive procedures to the pancreas, 2) a final diagnosis of probable or definitive type 1 AIP in accordance to the international consensus diagnostic criteria proposed in 2011 [2], 3) at least one month of follow-up with imaging modalities including CT, MRI, fluorine 18 fluorodeoxyglucose (FDG) positron emission tomography/CT (^18^F-FDG PET/CT) or ultrasonography (US) unless AIP and PaT were identified simultaneously. A total of 74 patients (56 from the first institution and 18 from the second institution) diagnosed with type 1 AIP were included (Fig. 1). The presence of PaT in AIP patients was confirmed histologically, and the absence of PaT in AIP patients was defined as no definite pathological or radiological evidence of PaT until the end of surveillance period.

### Clinical characteristics

**Fig. 2:**
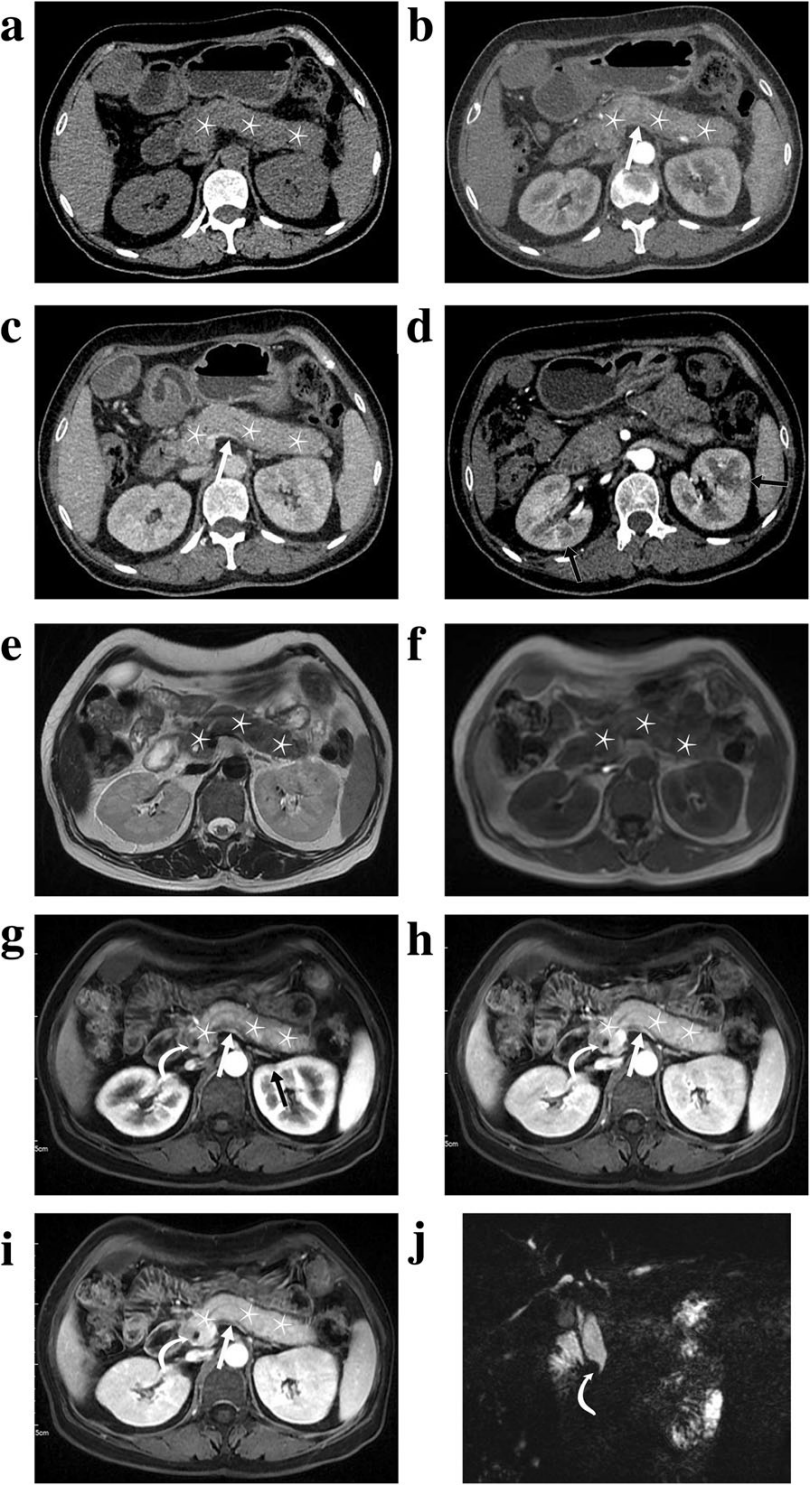
Typical CT and MR imaging appearance of type 1 autoimmune pancreatitis. Axial pre-enhanced (**a**), arterial (**b**), venous (**c**) phase on CT images and axial T2WI (**e**), T1WI (**f**), pancreatic (**g**), portal venous (**h**) and delayed venous (**i**) phase on MR imaging showed diffuse pancreatic enlargement with homogenous enhancement (star sign). On MR imaging, it appeared hypointense during the pancratic phase (**g**) compared with the spleen, and became hyperintense during the portal venous phase (**h**) compared with the pancreatic phase (**g**) and isointense during the delayed venous phase (**i**) compared with the portal venous phase (**h**). Peripancreatic vessel stricture (straight white arrows) was observed on both CT and MR images (**b**, **c**, **g**, **h** and **i**). Extrapancreatic involvement including the common bile duct (curved white arrows on **g**, **h** and **i**) and kidney (straight black arrows on **d** and **g**) were noted. The MR cholangiopancreatography (**j**) showed the stricture of the common bile duct and the dilatation of the upstream extrahepatic duct (curved white arrow)

The period of follow-up was calculated from the date of AIP recognition to that of most recent follow-up with imaging modalities or the date of PaT diagnosis in cases of concurrent PaT. Biopsy results at the initial visit were noted. The levels of serum IgG4 (normal range, ≤2 g/L) at the time of AIP diagnosis were categorized into within, 1–2 times and >2 times upper limit of normal value [2]. The absolute values of serum CA-199 (normal range, 0-35 U/ml) at the time of AIP diagnosis were also documented. Considering that different patients underwent examinations covering different body parts due to different physicians’ or patients’ preferences, the assessment of extrapancreatic involvement was limited to the typical organs involved in the abdomen, which included the bile ducts, kidneys, and retroperitoneum. In the absence of recent invasive procedures, neoplastic or infectious lesions in assessed organs, the extrapancreatic involvement was considered present on images if the bile ducts demonstrated thickening and contrast enhancement, the kidneys presented with round or wedge-shaped hypoattenuating lesions with moderate enhancement on contrast enhanced images, and the retroperitoneum was confined by a thick soft-tissue mass, respectively (Fig. 2) [5, 15].

### CT imaging characteristics

The pattern of pancreatic enlargement was recorded as either focal or diffuse. The presence or absence of pancreatic heterogeneity and low-density on enhanced images (arterial or venous phase), presence of pancreatic parenchymal atrophy, MPD dilatation (≥5 mm) [2] and cutoff, peripancreatic halo sign (capsule-like low-density rim), stranding, ascites and vessel stricture was evaluated (Fig. 2). If there were pre-existed hypodense areas in the pancreatic parenchyma, the shape, relative density and absolute density (Hounsfield, Hu) of low-density region were also recorded. The shape of hypodensity was noted as mass-like or non-mass-like. The relative density was noted as hypoattenuating, isoattenuating or hyperattenuating relative to the normal pancreatic parenchyma on arterial and venous phase images. A region of interest (ROI) was placed around the largest axial low-density region and drawn as large as possible excluding edge pixels to avoid partial volume effect. The mean absolute density values of ROIs (Hu) were separately measured on pre-enhanced, arterial and venous phase images.

### MR imaging characteristics

If patients also underwent MR before invasive procedures, the following MRI characteristics were assessed: 1) the pattern of pancreatic parenchyma involvement (focal or diffuse); 2) pancreatic parenchymal atrophy (present or absent); 3) the signal intensity of pancreatic parenchyma (hypo-, iso-, or hyperintense; homogenous or heterogeneous) compared with the unaffected pancreatic parenchyma or the liver on T1- and T2-weighted images; 4) contrast enhancement of pancreatic parenchyma (hypo-, iso-, or hyperintense; homogenous or heterogeneous) during the pancreatic phase compared with the unaffected pancreatic parenchyma or the spleen; 5) contrast enhancement of pancreatic parenchyma (hypo-, iso-, or hyperintense; homogenous or heterogeneous) during the portal venous phase compared with the pancreatic phase; 6) contrast enhancement of pancreatic parenchyma (hypo-, iso-, or hyperintense; homogenous or heterogeneous) during the delayed venous phase compared with the portal venous phase [17]; 7) rim enhancement of parenchyma on enhanced sequences (present or absent); 8) MPD dilatation (≥5 mm) [2] and cutoff (present or absent); 9) peripancreatic halo sign (capsule-like hyperintensity on T2WI and enhancement on delayed venous sequences) [18], stranding, ascites and vessel stricture (present or absent) (Fig. 2).

### Statistical analysis

Statistical analysis was performed with IBM SPSS Statistics software (version 21.0 J; SPSS, Chicago, IL). The Fisher’s exact test was used for qualitative variables, and nonparametric Mann-Whitney test for quantitative variables. A *p* value ≤0.05 was considered statistically significant.

## Results

### Characteristics of type 1 AIP patients with PaT

Of the included 74 type 1 AIP patients, 5 (6.7%,) were found to have the concurrent PaT. 3 cases (5.4%) were from the first institution and 2 (11.1%) from the second institution. The subtypes of the concurrent PaT were pancreatic ductal adenocarcinoma (PDAC) (3/5), solitary extramedullary plasmacytoma (SEP) in the pancreas (1/5) and cholangiocarcinoma (CC) in the pancreatic segment (1/5). Two patients (PDAC and CC) underwent pancreatic resection, and the remaining 3 cases were confirmed on the biopsy. The sizes of PADC and CC measured in specimen were 43 mm × 35 mm × 30 mm and 20 mm × 15 mm × 10 mm (compared with size of 43 mm × 32 mm × 29 mm and 18 mm × 14 mm × 12 mm measured on CT), respectively. The invasion to peripancreatic vessel was observed in the resected PDAC case, and the invasion to duodenum was noted in CC case. No metastases to lymph nodes were histologically noted in the two resected cases. The clinical and CT imaging characteristics of the 5 AIP patients with PaT were summarized in Table 1.

**Table 1:** Summary of Clinical and CT Imaging Characteristics of Type 1 AIP Patients with PaT

		PDAC (*n* = 3)		SEP (*n* = 1)	CC^a^ (n = 1)
	Case 1^a^	Case 2	Case 3		
Gender	Male	Male	Female	Female	Female
Surveillance period (month)	3	0	0	3	6
IgG4 (≤2 g/L, g/L)	6.9	5.2	4.6	0.3	4.1
CA-199 (0-35 U/ml, U/ml)	64.7	14.6	106.0	2.0	21.4
Biopsy at initial visit					
Location	Pancreas	Pancreas	Pancreas	Pancreas	Pancreas
Evidence of PaT	No	Yes	Yes	No	No
Presence of extrapancreatic involvement					
Bile duct	No	No	Yes	Yes	Yes
Kidney	No	No	No	No	No
Retroperitoneum	No	Yes	No	Yes	No
CT imaging characteristics of pancreatic parenchyma					
Focal enlargement	Yes	Yes	Yes	Yes	Yes
Presence of atrophy	No	No	Yes	No	No
Presence of heterogeneity	Yes	Yes	Yes	Yes	Yes
Presence of low-density	Yes	Yes	Yes	Yes	Yes
Mass-like	Yes	Yes	Yes	No	Yes
Relative density					
Arterial phase	Hypo	Hypo	Hypo	Hypo	Hypo
Venous phase	Hypo	Hypo	Hypo	Hypo	Iso
Absolute density (Hu)					
Pre-enhanced phase	38	45	28	45	45
Arterial phase	53	51	32	76	89
Venous phase	68	81	37	94	94
CT imaging characteristics of main pancreatic duct					
Dilatation	Yes	No	No	No	No
Cutoff	Yes	No	Yes	No	No
CT imaging characteristics of peripancreatic structures					
Presence of halo sign	Yes	No	No	Yes	No
Presence of stranding	Yes	No	Yes	Yes	No
Presence of ascites	No	No	No	Yes	No
Presence of vessel stricture	Yes	Yes	Yes	Yes	No

*Abbreviations*: *AIP* autoimmune pancreatitis, *PaT* pancreatic tumor, *PDAC* pancreatic ductal adenocarcinoma, *SEP* solitary extramedullary plasmacytoma, *CC* cholangiocarcinoma, *Hypo* hypoattenuating, *Iso* Isoattenuating, *Hu* Hounsfield

^a^ Marked cases underwent pancreatic resection, and the presence of concurrent PaT in other unmarked cases were proved by biopsy at the initial visit or in the surveillance period

**Fig. 3:**
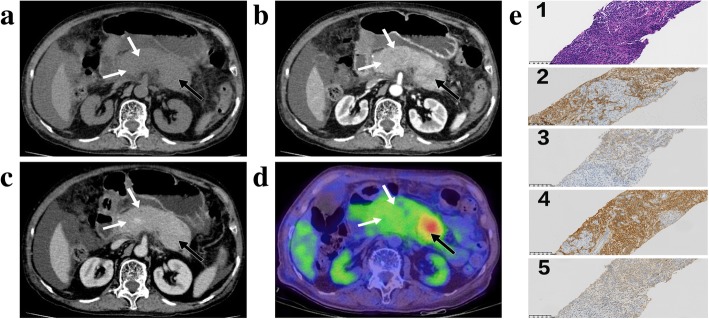
Type 1 autoimmune pancreatitis (AIP) with concurrent pancreatic solitary extramedullary plasmacytoma (SEP). Several areas of non-mass-like low-density (white and black arrows) in pancreas were found on arterial phase CT image (**b**). They appeared hypoattenuating on pre-enhanced (**a**) and venous (**c**) phase CT images. It was hard to differentiate SEP (black arrow) from the underlying AIP (white arrow) based on CT imaging findings (**a**, **b** and **c**). Fused fluorine 18 fluorodeoxyglucose (FDG) positron emission tomography/CT (^18^F-FDG PET/CT) (**d**) showed focal avid FDG uptake (black arrow) in the background of moderate FDG uptake (white arrow), which indicated there were two different entities present in the pancreas. The second biopsy of pancreas in the surveillance period showed dense infiltration of plasmacytoid cells on haematoxylin-eosin stain, original magnification × 200 (e-1). The plasmacytoid cells showed positive staining for CD138 (e-2), CD38 (e-3) and monotypic k-light chain (e-4), and negative staining for monotypic λ-light chain(e-5) on immunohistochemical stain, original magnification × 200

Two cases of PDAC were suspected due to the presentation of a mass-like hypodensity on the pancreatic head on CT scans, which were confirmed by the endoscopic ultrasonography-guided fine needle aspiration (EUS-FNA) of pancreas at the initial visit. They were recognized at the same time of AIP diagnosis, so the surveillance period were noted as 0 month. The presences of SEP, CC and the third PDAC case were suspected due to progression of size identified on follow-up CT. The surveillance periods were 3, 6 and 3 months, respectively. Notably, all three patients underwent EUS-FNA of pancreas initially, for which the results showed no evidence of tumor. The AIP patient with SEP underwent ^18^F-FDG PET/CT after the follow-up abnormal CT. The result of ^18^F-FDG PET/CT indicated the concurrent presence of a different entity from the underlying AIP, where a focal avid FDG uptake in the background of moderate FDG uptake was evident in the pancreas (Fig. 3).

### Comparison of clinical characteristics

Gender (*p* = 0.044) was a statistically significant factor that differed between AIP patients with and without PaT. 3 of 5 AIP patients with PaT were females. The levels of serum IgG4 and CA-199 were separately measured in 65 patients. There was no significant difference in respect to age, surveillance period, inclusion of biopsy, serum IgG4 or CA-199 levels, or extrapancreatic involvement between the two groups (*p*>0.05) (Table 2).

**Table 2:** Comparison of Clinical and CT imaging Characteristics between Type 1 AIP Patients with and without PaT

	AIP (*n* = 69)	AIP + PaC (*n* = 5)	*P* Value
Male	58 (84%)	2 (40%)	0.044
Age (year)	61 (24–80)	58 (42–76)	0.845
Surveillance period (month)	5 (1–109)	3 (0–6)	0.086
IgG4 (*n* = 65)			0.561
<1 time upper limit	8 (14%)	1 (20%)	
1–2 times upper limit	11 (17%)	0 (0%)	
≥ 2 times upper limit	41 (69%)	4 (80%)	
CA-199 (n = 65, U/ml)	19.5 (2.0–1987.0)	21.4 (2.0–106.0)	0.767
Biopsy at the initial visit	41 (58%)	5 (100%)	0.150
Presence of extrapancreatic involvement			
Bile duct	63 (91%)	3 (60%)	0.087
Kidney	25 (36%)	0 (0%)	0.160
Retroperitoneum	9 (9%)	2 (40%)	0.157
CT imaging characteristics of pancreatic parenchyma			
Enlargement pattern			0.003
Diffuse	49 (71%)	0 (0%)	
Focal	20 (29%)	5 (100%)	
Presence of atrophy	10 (15%)	1 (20%)	0.564
Presence of heterogeneity	28 (41%)	5 (100%)	0.015
Presence of low-density (*n* = 26)	21 (30%)	5 (100%)	0.004
Mass-like low-density	6 (29%)	4 (80%)	0.055
Relative density of low-density			
Arterial phase			1.000
Hypoattenuating	21 (100%)	5 (100%)	
Isoattenuating	0 (0%)	0 (0%)	
Hyperattenuating	0 (0%)	0 (0%)	
Venous phase			0.562
Hypoattenuating	14 (67%)	4 (80%)	
Isoattenuating	7 (33%)	1 (20%)	
Hyperattenuating	0 (0%)	0 (0%)	
Absolute density of low-density (Hu)			
Pre-enhanced phase	42 (33–53)	45 (28–45)	0.769
Arterial phase	71 (51–96)	53 (32–89)	0.313
Venous phase	83 (57–112)	81 (37–94)	0.255
CT imaging characteristics of main pancreatic duct			
Presence of dilatation	16 (23%)	1 (20%)	1.000
Presence of cutoff	31 (45%)	2 (40%)	1.000
CT imaging characteristics of peripancreatic structures			
Presence of halo sign	43 (62%)	2 (40%)	0.374
Presence of stranding	14 (20%)	3 (60%)	0.076
Presence of ascites	2 (3%)	1 (20%)	0.192
Presence of vessel stricture	44 (64%)	4 (80%)	0.651

*Abbreviations*: *AIP* autoimmune pancreatitis, *PaT* pancreatic tumor, *Hu* Hounsfield

Quantitative data are expressed as median (range), qualitative data as absolute values (percentages)

### Comparison of CT imaging characteristics

**Fig. 4:**
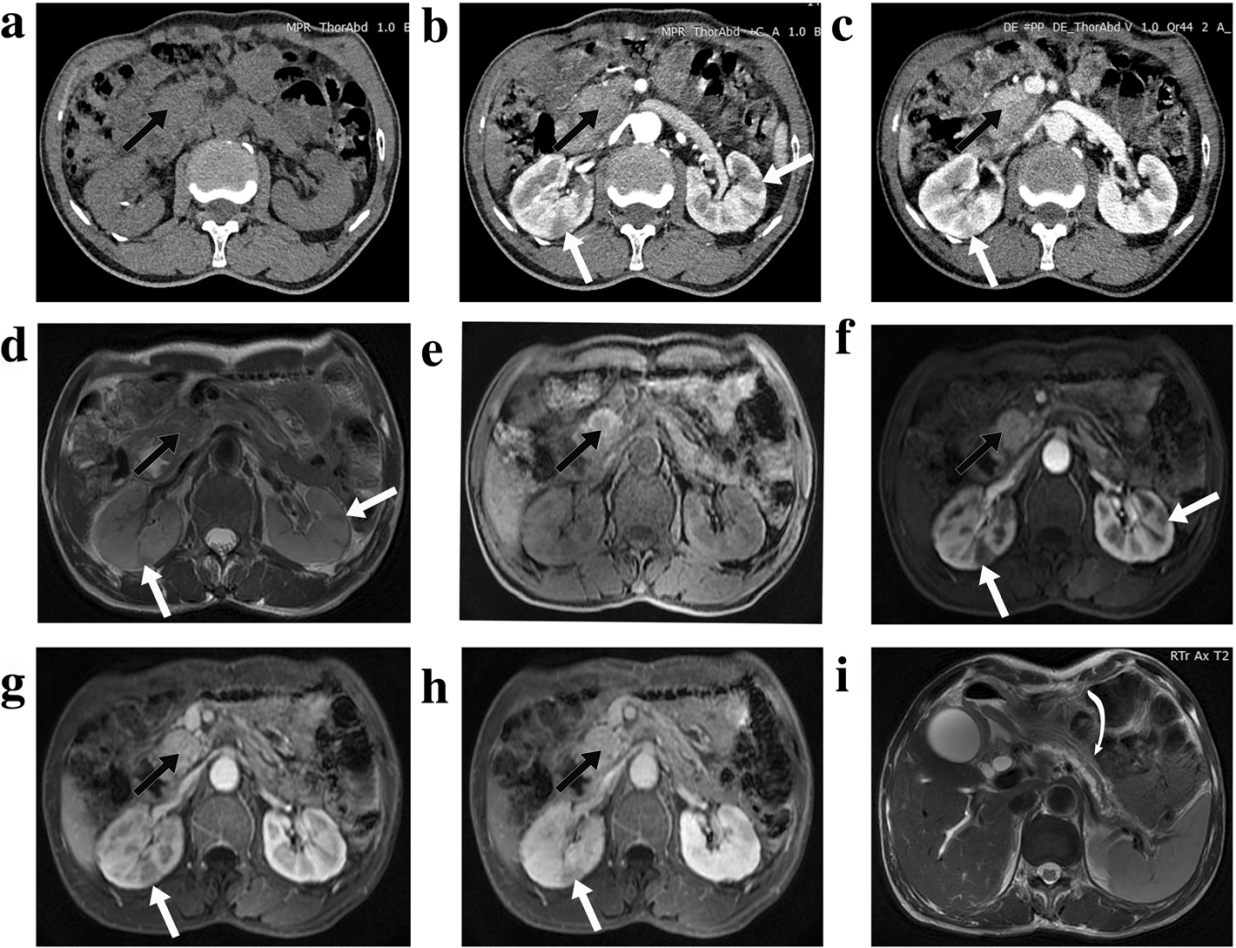
Type 1 autoimmune pancreatitis (AIP) without concurrent pancreatic tumor. Axial pre-enhanced (**a**), arterial (**b**), venous (**c**) phase on CT and axial T2WI (**d**), T1WI (**e**), pancreatic (**f**), portal venous (**g**) and delayed venous (**h**) phase on MR imaging showed a focal lesion in the pancreatic head (straight black arrows). It presented with mass-like low-density on enhanced CT images (**b** and **c**). MR images showed a focal lesion with nearly homogeneous enhancement on enhanced sequences (**f**, **g** and **h**). The presence of focal mass-like lesion, combined with the characteristics of distal pancreatic parenchymal atrophy and main pancreatic ductal dilatation (curved white arrow on **i**), led to the high suspicion of pancreatic tumor. This patient finally underwent pancreatic resection and was proved to be pure AIP histologically. Kidney involvement was also observed on both CT and MR imaging (straight white arrows on **b**, **c**, **d**, **f**, **g** and **h**)

The pattern of pancreatic enlargement (*p* = 0.003), the presence of pancreatic heterogeneity (*p* = 0.015) and low-density (*p* = 0.004) were significantly different between AIP patients with and without PaT. All of the 5 AIP patients with concurrent PaT demonstrated the CT imaging features of focal pancreatic enlargement, pancreatic heterogeneity and low-density (Table 2). Of note, there were 3 pure AIP patients (2 of 3 from the first medical institution) who presented with focal pancreatic enlargement, heterogeneity and mass-like low-density on CT in our cohort (Fig. 4). All of them eventually underwent pancreatic resection due to high suspicion of concurrent PaT. Other CT imaging characteristics of pancreatic parenchyma, MPD or peripancreatic structures did not differ significantly between the two groups (p>0.05) (Table 2). None of the low-density imaging characteristics on CT could significantly differentiate the two groups (*p*>0.05) (Table 2).

### Comparison of MR imaging characteristics

**Fig. 5:**
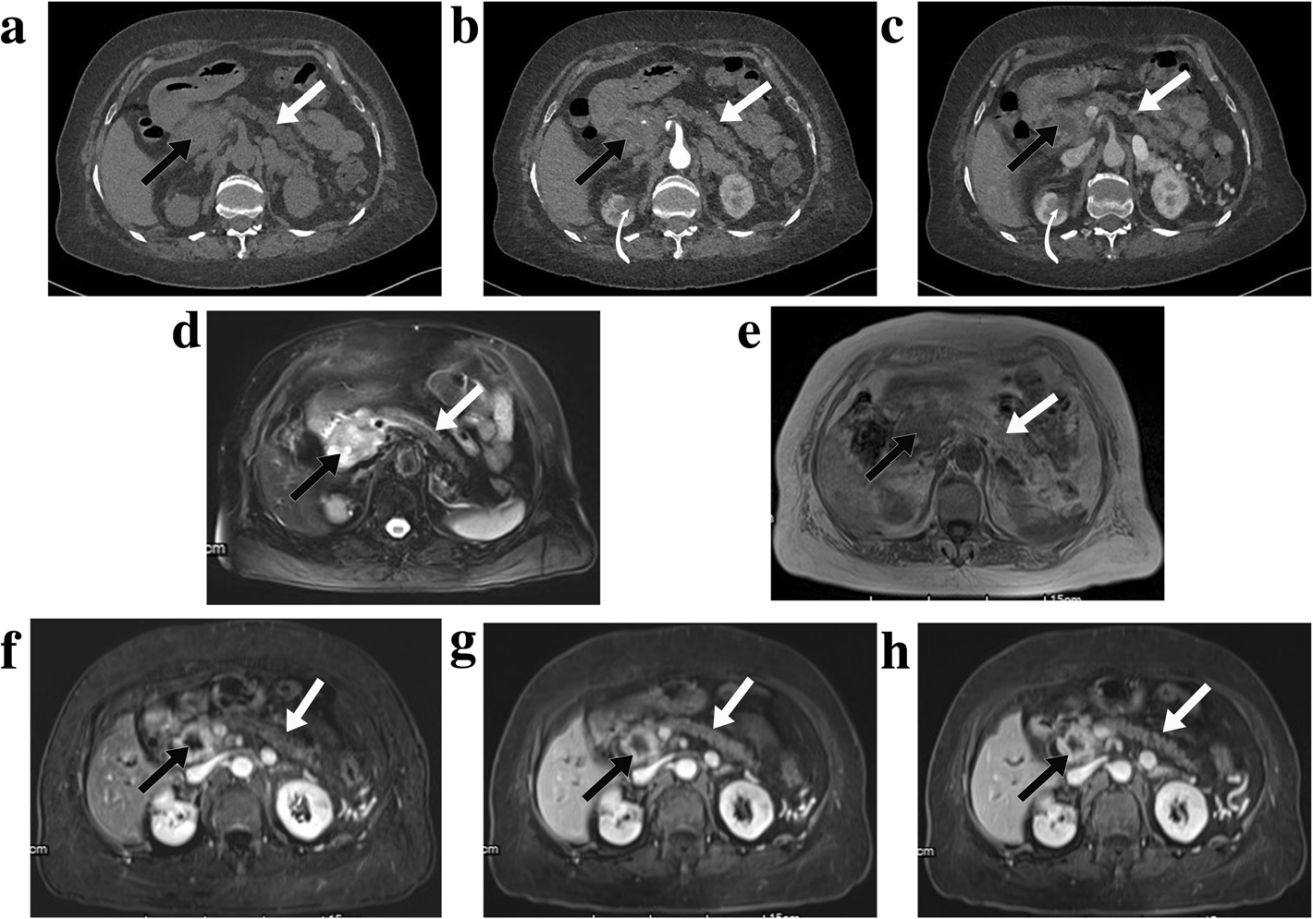
Type 1 autoimmune pancreatitis with concurrent pancreatic ductal adenocarcinoma. Axial pre-enhanced (**a**), arterial (**b**), venous (**c**) phase on CT and axial T2WI (**d**), T1WI (**e**), pancreatic (**f**), portal venous (**g**) and delayed venous (**h**) phase on MR imaging showed a focal lesion in the pancreatic head (straight black arrows). It appeared as low-density on enhanced CT images (**b** and **c**). The imaging characteristic of rim enhancement was noted on enhanced MR images (**f**, **g** and **h**), which was not obvious on CT. Distal pancreatic parenchymal atrophy was observed (straight white arrows on **a**-**h**). Kidney involvement was also seen (curved white arrows on **b** and **c**)

Among the included patients, 20 patients had the available MR imaging data that were analyzed. Only one case (PDAC from the first medical center) was from the AIP + PaT group, which appeared as a focal lesion with rim enhancement (Fig. 5). None of the pure AIP cases showed the feature of rim enhancement, even in the cases with focal pancreatic enlargement (Fig. 4). It differed significantly between the comparing groups (*p* = 0.050). None of the other evaluated MR imaging characteristics worked significantly (*p*>0.05) (Table 3).

**Table 3:** Comparison of MR imaging Characteristics between Type 1 AIP Patients with and without PaT

	AIP (*n* = 19)	AIP + PaC (n = 1)	*P* Value
Pancreatic parenchyma			
Enlargement pattern			0.250
Diffuse	15 (79%)	0 (0%)	
Focal	4 (21%)	1 (100%)	
Presence of atrophy	2 (11%)	1 (100%)	0.150
Signal intensity on T1WI			
Hypointense	16 (84%)	1 (100%)	0.911
Isointense	2 (11%)	0 (0%)	
Hyperintense	1 (5%)	0 (0%)	
Presence of heterogeneity	5 (26%)	0 (0%)	1.000
Signal intensity on T2WI			
Hypointense	0 (0%)	0 (0%)	0.452
Isointense	7 (37%)	0 (0%)	
Hyperintense	12 (63%)	1 (100%)	
Presence of heterogeneity	6 (31%)	1 (100%)	0.300
Signal intensity on pancreatic phase			
Hypointense	13 (69%)	1 (100%)	0.502
Isointense	6 (31%)	0 (0%)	
Hyperintense	0 (0%)	0 (0%)	
Presence of heterogeneity	12 (63%)	1 (100%)	1.000
Signal intensity on portal venous phase			
Hypointense	1 (5%)	0 (0%)	0.798
Isointense	5 (26%)	0 (100%)	
Hyperintense	13 (69%)	1 (0%)	
Presence of heterogeneity	6 (31%)	1 (100%)	0.350
Signal intensity on delayed venous phase			
Hypointense	3 (16%)	0 (0%)	0.753
Isointense	12 (63%)	1 (100%)	
Hyperintense	4 (21%)	0 (0%)	
Presence of heterogeneity	5 (26%)	1 (100%)	0.300
Presence of rim enhancement	0 (0%)	1 (100%)	0.050
Main pancreatic duct			
Presence of dilatation	3 (16%)	0 (0%)	1.000
Presence of cutoff	6 (31%)	1 (100%)	0.350
Peripancreatic structures			
Presence of halo sign	1 (5%)	0 (0%)	1.000
Presence of stranding	0 (0%)	0 (0%)	1.000
Presence of ascites	0 (0%)	0 (0%)	1.000
Presence of vessel stricture	13 (69%)	1 (100%)	1.000

*Abbreviations*: *AIP* autoimmune pancreatitis, *PaT* pancreatic tumor

Data are expressed as absolute values (percentages)

## Discussion

In the present study, we found that the gender, pattern of pancreatic enlargement, pancreatic heterogeneity, low-density on enhanced CT and rim enhancement on MRI were significantly different between type 1 AIP patients with and without PaT. 3 of 5 AIP + PaT patients were females. All of them demonstrated focal pancreatic enlargement, heterogeneity and low-density on CT. The details of low-density on CT didn’t work significantly. Only one AIP + PcT patient had the MRI data, and showed the characteristic of rim enhancement. None of the pure AIP patients presented with rim enhancement on MR imaging. According to our knowledge, there was only one comparable study that has attempted to identify the difference between type 1 AIP patients with and without PaT, but no statistically significant differences were concluded [8].

The prevalence of PaT in AIP patients was about 5.4% (3/56) in the first medical institution and 11.1% (2/18) in the second medical institution. The general prevalence of concurrent PaT in type 1 AIP in the two institutions was about 6.7% (5/74), which was higher than other studies [5–9, 11, 12]. The reason for the higher prevalence of PaT in AIP patients, especially in the second medical institution, may be a result of potential selection bias. The second institution is a cancer specific center, where non-cancer patients such as pure AIP cases may not be referred for surveillance. The definite association between AIP and PaT remained inconclusive. Some researchers have suggested that AIP may be a manifestation of paraneoplastic syndrome [10, 16], some have indicated that chronic pancreatic inflammation in AIP may contribute to carcinogenesis [8, 12, 19], and some have reported that AIP was not associated with an increased incidence of total malignancies [20, 21]. Our results tended to support the hypothesis of paraneoplastic syndrome, which would be briefly explained below.

The concurrent PaTs were identified 0, 3 and 6 months after the diagnosis of AIP, respectively. But during retrospective review, all of the PaTs were concurrently present with AIP (this synchronous occurrence may indicate the association between AIP and paraneoplastic syndrome). Several studies have reported that the occurrence of cancer in AIP patients was significantly higher in the first year than in the subsequent years [12, 16]. Accordingly, some PaTs were identified at the same time of AIP diagnosis and some were found during the follow-up period that could last as long as 186 months [7–9, 12, 22, 23]. Therefore optimal surveillance duration in AIP patients cannot be concluded for early PaT detection. However, close observation should be warranted during the first year post diagnosis of AIP given the diagnostic challenge and relatively high incidence during the first year. In this cohort, the delay in diagnosing the concurrent PaT was mainly attributed to the negative EUS-FNA results of pancreas at the initial visit. It indicated that the negative EUS-FNA results of pancreas couldn’t rule out the presence of PaT, which was consistent with the results of some previous studies [19, 24]. Several studies showed that serum IgG4 levels at the diagnosis of AIP were significantly higher in AIP patients with cancer than those without cancer [12, 16]. In our study, the serum IgG4 level in 4 of 5 AIP + PaT patients went beyond the 2 times upper limit of normal value, though it didn’t differ significantly between AIP patients with and without PaT. The types of included cancers in the previous studies were mainly non-PaT tumors [12, 16], it remains questionable whether different types of concurrent cancer would result in different levels of serum IgG4 elevation. Age, serum CA-199 levels or extrapancreatic involvements did not vary significantly between the two groups, which were consistent with previous studies [8, 12]. In the current study, the only significant clinical characteristic was gender and 3 of 5 AIP patients with PaT were females. Comparably, Ikeura et al. found that 2 out of 3 AIP patients with PaT were females, though gender difference between the two groups didn’t reach statistical significance [8]. However, there were previous studies where the reported AIP + PaT patients were all males [9, 19]. The reason for the discrepancy between our and previous study is unknown. This may be due to the limited sample size in both current and previous studies. The female AIP + PaT predominance in our cohort may suggest that at least some AIP cases are associated with paraneoplastic syndrome. When type 1 AIP appears in an uncommon population, intense attention should be paid to exclude the concurrent presence of a tumor.

The CT imaging characteristics of focal pancreatic enlargement, pancreatic heterogeneity and low-density on enhanced phase were significantly different between AIP patients with and without PaT in this cohort. All of the three CT imaging features were detected in all of the 5 cases with accompanied PaT. Comparably, Ikeura et al. also found that all of the 3 AIP patients who developed PaT showed the feature of focal pancreatic enlargement, but this characteristic didn’t differ significantly between the comparison groups [8]. When the inclusion criteria was set as focal pancreatic enlargement that underwent resection, the incidence of concurrent PaT in type 1 AIP could be as high as 33.3% (2/6) [19], which was much higher than previously mentioned. Besides, Ikeura et al. found 2 out of 3 AIP + PaT patients presented with pancreatic low-density, but statistical significance was not provided [8]. The international consensus considers pancreatic low-density as an atypical imaging finding of AIP and suggests such patients should be managed as PaT unless an alternative diagnosis is strongly suspected [2]. Therefore concurrent pancreatic malignancy needs to be excluded in the presence of focal pancreatic enlargement and low-density in AIP. Notably, focal pancreatic enlargement, pancreatic heterogeneity and low-density did appear in pure AIP cases as we have shown, so the presence of these features do not validate concurrent PaT. Furthermore, the diagnosis cannot be further made easier by the interpretation of other CT imaging characteristics. 4 of 5 AIP patients with accompanied PaT presented with mass-like low-density despite no statistically significant difference was observed between AIP patients with and without PaT. Notably, the shape of mass-like low-density was also noted in small portions of pure AIP cases (6/21). Our results showed that the sizes of concurrent PaT measured in specimen were comparable with initial CT measurement, which indicated the presence of mass-like low-density was highly suggestive of concurrent tumor. However, when the tumor was too small to exceed the contour of pancreas on imaging, it was hard to conclude the presence of concurrent PaT. In this situation, close surveillance was of vital importance, which was supported by the fact that 3 cases of AIP + PaT became highly suspicious for malignancy during the follow-up period in our cohort. On the other hand, if the PaT was very large and appeared obvious in the background of pancreas on imaging, the diagnosis of AIP + PaT may be easy to make. Consequently, the morphologic criteria should be modified with the stage or the size of the tumor. The effect of size difference between pathology and radiology on the interpretation of mass-like low-density needs future investigations to explore, where larger sample size are warranted. In this cohort, the low-density areas in both AIP and PaT portions presented as hypoattenuating region during arterial phase and either hypoattenuating or isoattenuating during venous phase. There was not statistically significant absolute density value identified to indicate the underlying pathological differences between the two. So the presence of pancreatic low-density regions on CT in patients with AIP was only suggestive for the presence of concurrent PaT. Other imaging characteristics suggestive of PaT include MPD dilatation, pancreatic parenchymal atrophy and peripancreatic vessel stricture. However, these characteristics were also noted in pure AIP cases, which was in accordance with previous studies [3, 14, 25]. These imaging features in AIP may be attributed to the longstanding intra- and peri-pancreatic inflammation and fibrosis in AIP.

In the aspect of MR imaging characteristics, our results showed that AIP + PaT patient could present with rim enhancement on MR imaging, and none of the included pure AIP cases showed this imaging characteristic. So it may be a useful MR imaging characteristic to differentiate pure AIP cases from AIP + PaT cases. However, neither the role of MR imaging characteristics in differentiating AIP patients with and without PaT nor the presence of rim enchantment on MR imaging in pure AIP cases has ever been studied previously [17, 18]. Moreover, there was only one AIP + PaT case included in the present cohort. So it is still too early to draw a final conclusion on the role of MR imaging in differentiating the two groups, and future studies with larger sample size are recommended.

Besides CT and MR, the case of AIP with SEP in our study implies that ^18^F-FDG PET/CT may add some value where the difference in FDG uptake may help identify the concurrent PaT. However, Shiokawa et al. [16] reported that none of AIP patients with cancer were identified with ^18^F-FDG PET/CT, though there was no case of PaT in their cohort. Further studies are required before definitive conclusion can be made.

There are limitations to our study. Firstly, though we have included the AIP + PaT data of two institutions, only a small size of cases was identified due to its rare entity. Secondly, the AIP patients in the two medical institutions follwed different diagnostic and follow-up pathways due to various reasons where different imaging modalities were used for surveillance which further limited the sample size as some did not meet the selection criteria.

## Conclusion

When type 1 AIP patients are females and present with focal pancreatic enlargement, pancreatic heterogeneity, low-density on CT and rim enhancement on MR imaging, it should be prudent to exclude the synchronous presence of PaT. The CT imaging characteristics of pancreatic low-density or other MR imaging characteristics of intra−/peri-pancreatic structures are not reliable to conclude the presence of concurrent PaT.

## Data Availability

The datasets used and analyzed during the current study are available from the corresponding author on reasonable request.

## Abbreviations

^18^F-FDG PET/CT: fluorine 18 fluorodeoxyglucose positron emission tomography/CT
AIP: Autoimmune pancreatitis
CC: Cholangiocarcinoma
EUS-FNA: Endoscopic ultrasonography-guided fine needle aspiration
MPD: Main pancreatic duct
PaT: Pancreatic tumor
PDAC: Pancreatic ductal adenocarcinoma
SEP: Solitary extramedullary plasmacytoma
US: Ultrasonography
